# Characterization of 15 nuclear microsatellite markers for *Afzelia africana* (Fabaceae) and related species

**DOI:** 10.1002/aps3.1249

**Published:** 2019-05-13

**Authors:** Thierry D. Houehanou, Kathleen Prinz, Frank Hellwig

**Affiliations:** ^1^ Laboratory of Applied Ecology Faculty of Agricultural Sciences University of Abomey 01 BP 526 Cotonou Republic of Benin; ^2^ Institute for Ecology and Evolution Professorship for Systematic Botany with Hausknecht Herbarium and Botanical Garden Friedrich Schiller University Jena 07743 Jena Germany; ^3^ Laboratory of Ecology, Botany and Plant Biology Faculty of Agronomy University of Parakou 03 BP 125 Parakou Republic of Benin; ^4^ Laboratoire de Biomathématiques et d'Estimations Forestières Faculté des Sciences Agronomiques Université d'Abomey Calavi 04 BP 1525 Cotonou Republic of Benin; ^5^ Landschaftspflegeverband Suedharz/Kyffhaeuser e.V. 99734 Nordhausen Germany

**Keywords:** *Afzelia africana*, conservation genetics, Fabaceae, simple sequence repeat (SSR), sustainable management, West Africa

## Abstract

**Premise:**

*Afzelia africana* (Fabaceae) is a valuable, internationally vulnerable tree species in tropical Africa. The development of specific simple sequence repeat (SSR) loci is necessary for population genetic studies in this tree species and its closest relatives.

**Methods And Results:**

Fifteen new polymorphic microsatellite markers were developed for *A. africana* using Illumina next‐generation sequencing. We tested the polymorphism of the 15 loci in three populations in Benin, West Africa. The number of expressed alleles per locus varied from one to 12. The levels of observed and expected heterozygosity ranged from 0.100 to 1.000 and from 0.095 to 0.882, respectively. Most markers successfully amplified in the closely related species *A. quanzensis* and *A. bipindensis*, but less so in *A. bipindensis*.

**Conclusions:**

Because of their cross‐amplification ability, these newly developed loci will serve as useful tools for future molecular analyses on *A. africana* and related species.

*Afzelia africana* Sm. (Fabaceae) is a valuable tree species distributed in tropical Africa from Senegal to Tanzania. The species is highly exploited by local communities due to its value in medicine, fodder, and wood for fuel and tools (Houehanou et al., 2011). Because of its multiple uses, a serious decline in natural populations has been reported in many places, especially in West Africa (Hahn‐Hadjali and Thiombiano, 2000; Adomou et al., 2009). Consequently, *A*. *africana* is listed as a vulnerable tree species on the International Union for Conservation of Nature (IUCN) Red List (African Regional Workshop, 1998). In West Africa, much research effort has been invested in promoting the sustainable management and conservation of the species by assessing the local importance of its uses (Houehanou et al., 2011; Balima et al., 2018) and the variation of its population attributes in relation to climatic condition and anthropogenic disturbance (Nacoulma et al., 2011; Mensah et al., 2014; Amahowe et al., 2018). However, little is known of the population genetics of *A. africana*, as is the case for most endangered African tree species (Houehanou et al., 2014). At present, nuclear microsatellite markers are the most significant molecular tools in developing a deeper understanding of processes such as genetic diversity, inbreeding, and gene flow that affect sustainable management and conservation. Nuclear microsatellite markers are highly polymorphic, mostly species specific, and the transferability of these markers among closely related species is possible. However, transferring these markers from one species to a closely related one is often accompanied by decreased polymorphism, which limits the assessment of their genetic diversity and the detection of potential differentiation among populations. Nuclear microsatellite loci, to date, are available for the related species *A. bipindensis* Harms (Donkpegan et al., 2015) and *A. quanzensis* Welw. (Jinga et al., 2016). Nuclear microsatellite markers, such as specific simple sequence repeat (SSR) markers, are transferable but not specific to *A. africana*. Tests of suitability in *A. africana* showed less polymorphism for most of the SSRs developed so far (Houehanou et al., unpublished data). Only three SSR loci are therefore suitable for population genetic studies in the target species (Houehanou et al., unpublished data). Hence, we developed and established a new set of 15 polymorphic SSR microsatellite loci specific to *A. africana* to initiate meaningful population genetic studies of this tree species.

## METHODS AND RESULTS

DNA was extracted from silica‐dried leaf material using a modified cetyltrimethylammonium bromide (CTAB) protocol (Doyle and Doyle, 1987). For development of microsatellite loci, genomic DNA was isolated from one individual of *A. africana* located in the Lama Forest, Benin (geographic coordinates: 6.976576°N, 2.135348°E). A specimen (Houehanou 024) of this individual was prepared and stored at the Institute for Ecology and Evolution, Friedrich Schiller University, Jena, Germany. Additionally, a herbarium specimen (YH246) from the same locality (Lama Forest) was prepared and deposited at the herbarium of the University of Abomey Calavi (Appendix 1).

Library preparation, enrichment, sequencing, and detection of microsatellite loci were performed by AllGenetics (A Coruña, Spain). DNA was fragmented and tagged using tagmentase (Illumina, San Diego, California, USA) with the reaction mix incubated in a thermal cycler at 55°C for 5 min. The reaction was neutralized with 5 μL of Neutralize Tagment Buffer (Illumina). Tagmented DNA was subjected to one round of PCR in a Bio‐Rad iCycler (Bio‐Rad, Hercules, California, USA), in which indexes and Illumina adapters were added to the ends of each of the fragments. The PCR was performed under the following conditions: an initial incubation at 72°C for 3 min; denaturation at 95°C for 30 s; followed by 12 cycles of 95°C for 10 s, 55°C for 30 s, and 72°C for 30 s; with a final elongation step at 72°C for 5 min. The amplified library was purified using 30 μL of AMPure XP beads (Beckman Coulter, Brea, California, USA). The purified library was enriched for microsatellite motifs by hybridizing the biotin‐tagged oligonucleotide probes to the library molecules. The fragments containing microsatellite motifs were recovered by adding 100 μL of Dynabeads M‐280 (Invitrogen, Carlsbad, California, USA) and the use of a magnet. The enriched pool was subjected to a final PCR round using the proofread enzyme Phusion HiFi Master Mix (Thermo Fisher Scientific, Waltham, Massachusetts, USA). The PCR conditions were as follows: an initial denaturation at 98°C for 30 s; followed by 30 cycles of 98°C for 10 s, 65°C for 20 s, and 72°C for 20 s; and a final extension step at 72°C for 5 min. The PCR product was purified using 30 μL of AMPure XP beads.

Enriched libraries were sequenced using the Illumina MiSeq platform (paired‐end, 300‐bp reads), producing 3,751,572 raw reads. These reads were processed and filtered in Geneious 10.0.9 (Biomatters Ltd., Auckland, New Zealand) as follows. Reads were trimmed using an error probability limit of 0.03. Merging of the forward (R1) and reverse (R2) reads was performed with FLASH (Magoc and Salzberg, 2011). Mismatch resolution in the overlapping region (minimum overlap of 50 bp) was accomplished by keeping the base with the higher quality score. The raw data were deposited at the National Center for Biotechnology Information (NCBI) Sequence Read Archive (accession number SRP172013).

Five hundred primer pairs were designed from the trimmed, merged reads using Primer3 (Koressaar and Remm, 2007; Untergasser et al., 2012) as implemented in Geneious 10.0.9. Default parameter values were used. From the 500 primer pairs, we selected the first 20 for screening following the quality criteria of Li et al. (2002). Specifically, primer pairs with two, three, and four or more bases per motif were selected with a minimum number of repeat motifs of 10, seven, and five, respectively. Primer pairs were tested for functionality and variability in a subset of 60 samples of *A. africana* collected from three populations in southern, central, and northern Benin (Appendix 1). PCR reactions were conducted with an Eppendorf Mastercycler ep gradient S thermocycler (Eppendorf, Hamburg, Germany) in a final volume of 10 μL with a final concentration of buffer of 25 mM MgCl_2_ (Thermo Fisher Scientific), 0.2 mM dNTPs (Thermo Fisher Scientific), 0.3 μM of each primer, 0.025 U/μL *Taq* polymerase (Thermo Fisher Scientific), and 0.2–1 ng/μL of template DNA. The PCR program included an initial denaturation at 94°C for 5 min; 35 cycles of denaturation at 94°C for 60 s, annealing at locus‐specific temperatures for 60 s, and elongation at 72°C for 60 s; and final elongation at 72°C for 20 min (Table 1). Touchdown PCR procedures were tested and used for some loci, implementing a reduction of 1°C per cycle within a locus‐specific temperature frame (Table 1). Amplification products were separated electrophoretically on 40% polyacrylamide sequencing gels using a LI‐COR Long Readir 4200 (Global Edition IR2 DNA Sequencer; Li‐Cor Biosciences, Lincoln, Nebraska, USA). Microsatellite fragments were scored by visual inspection using an internal 67–315‐bp DNA size standard.

**Table 1:** Characteristics of 15 microsatellite loci isolated from *Afzelia africana*

Locus	Primer sequences (5′–3′)	Repeat motif	*T*_a_ (°C)	TD (°C)	Allele size range (bp)	GenBank accession no.
AG_Aaf_12	F: GCTGAAAGGAGTGCAACACG	(AAT)_7_	60		133–145	MH814983
	R: GTTCGACGCTTGGGTATCGA					
AG_Aaf_14	F: AGAAAGCTGACCTCAACGCA	(AG)_14_	61		83–103	MH814984
	R: TAAACAGGTCCGTCCCTCCT					
AG_Aaf_15	F: GACATGCCTTCTTTCGCCAC	(AAAAC)_7_	59	66 → 56	127–142	MH814985
	R: GAGGTTGAGGCCTCGATGAG					
AG_Aaf_131	F: TTGGAGACGATCAGGACACG	(AG)_11_	57	63 → 55	288–294	MH814986
	R: TGCGATGCAGTTCCAACCT					
AG_Aaf_212	F: AGGGCCATTCAGACCTTTCT	(AG)_17_	55	64 → 54	169–195	MH814987
	R: GTCCTGGCCCAGATGACTAT					
AG_Aaf_214	F: AGCACGTCTTGTTGATGATGTC	(AAG)_22_	61	68 → 58	186–231	MH814988
	R: CGCAGTTGTAGCTATCCCGT					
AG_Aaf_276	F: AGATTTCCTCTCGTTGTAGCAGT	(AG)_12_	60		91–101	MH814989
	R: GGGTGAGTTGGAATACGGCT					
AG_Aaf_305	F: TAAGTGGCTCATGACCTCCG	(AG)_17_	55	65 → 53	83–111	MH814990
	R: CCTAGACATTCGGTGTTACACGT					
AG_Aaf_350	F: TCAAGTTTACTATGCTCGCCCT	(AG)_12_	60	67 → 57	113–135	MH814991
	R: ACTGAGCTGGTGTAGATGATCG					
AG_Aaf_358	F: TGCTTCCAATTCATTCAACTCGT	(ATC)_9_	65		85–103	MH814992
	R: ACCTCAACAACTGGTGATGCT					
AG_Aaf_386	F: TTACCAGAGGAAGGATTCTGCG	(AAG)_7_	55	65 → 53	237–240	MH814993
	R: TGAAGAAGGCGTTGATTGTTCA					
AG_Aaf_392	F: AGCAAATATCTCTTCACGCTTGT	(AG)_19_	60		73–91	MH814994
	R: ACAGCAAATGAAACAGAGGCG					
AG_Aaf_403	F: ATACCTCATCGGGCGGAGT	(ATATC)_5_	57	63 → 55	117–127	MH814995
	R: TGCGATTGTAGAAATGGCTAAGT					
AG_Aaf_422	F: ATCTTTCGTCGTAGAACCAAGG	(AG)_13_	58	64 → 56	87–97	MH814996
	R: TTTGTGTCCGTTCGTAAAGCA					
AG_Aaf_434	F: CGGATTGACTCTATTCAACTCCC	(AATGG)_5_	55	65 → 53	99–104	MH814997
	R: CCTGAGTGCAATGGAATGGAG					

Note

*T*_a_ = annealing temperature; TD = touchdown.

Of the 20 loci tested, 15 yielded unambiguously scorable and polymorphic products in *A. africana,* although one of the 15 loci was monomorphic in two populations (Table 2). The remaining five loci failed to amplify or yielded ambiguous bands. Loci were analyzed for number of alleles, levels of observed and expected heterozygosity, and Hardy–Weinberg equilibrium (HWE) using GenAlEx 6.5 (Peakall and Smouse, 2012). A chi‐square test was used to test for significant difference of HWE (α = 0.05), and various levels of significant differences were included (*P* < 0.05, *P* < 0.01, *P* < 0.001). Allele numbers per locus varied from one to nine for the northern population, two to 11 for the central population, and one to 12 for the southern population (Table 2). Levels of observed and expected heterozygosity ranged globally from 0.100 to 1.000 and from 0.095 to 0.882, respectively (Table 2). Several loci deviated from HWE (Table 2). Although the number of alleles per locus was not high, observed heterozygosity was somewhat higher than expected for some loci and was in the range suggested for outbreeding species. The loci that amplified successfully in *A. africana* samples were also tested for cross‐amplification in eight individuals of *A. quanzensis* and 10 individuals of *A. bipindensis* (Table 3). All 15 loci amplified in *A. quanzensis* but only 11 amplified in *A. bipindensis* (Table 3).

**Table 2:** Genetic diversity parameters for 15 polymorphic microsatellite loci in three populations of *Afzelia africana* in Benin, West Africa.^a^

Locus	Northern Benin (*n* = 20)				Central Benin (*n* = 20)				Southern Benin (*n* = 20)			
	*A*	*H*_o_	*H*_e_	HWE	*A*	*H*_o_	*H*_e_	HWE	*A*	*H*_o_	*H*_e_	HWE
AG_Aaf_12	3	0.857	0.584	**	2	0.789	0.494	**	3	1.000	0.611	**
AG_Aaf_14	9	1.000	0.820	ns	11	1.000	0.852	*	10	1.000	0.827	ns
AG_Aaf_15	2	0.867	0.500	**	4	0.632	0.737	*	4	0.688	0.725	***
AG_Aaf_131	4	1.000	0.584	*	4	0.800	0.640	***	2	0.647	0.493	ns
AG_Aaf_212	9	1.000	0.857	ns	10	0.944	0.861	ns	12	1.000	0.882	ns
AG_Aaf_214	9	0.882	0.754	ns	8	1.000	0.829	ns	8	0.846	0.775	**
AG_Aaf_276	4	0.647	0.507	ns	6	0.789	0.799	ns	5	1.000	0.727	ns
AG_Aaf_305	9	0.647	0.856	***	8	0.667	0.858	***	6	0.733	0.776	*
AG_Aaf_350	6	0.867	0.749	ns	11	0.842	0.877	ns	10	1.000	0.834	ns
AG_Aaf_358	5	0.733	0.549	ns	5	0.778	0.654	**	5	0.882	0.687	***
AG_Aaf_386	1	0.000	0.000	—	2	0.100	0.095	ns	1	0.000	0.000	‐
AG_Aaf_392	7	1.000	0.797	*	6	0.950	0.744	*	6	0.857	0.686	ns
AG_Aaf_403	3	0.688	0.471	ns	3	0.900	0.545	**	3	0.750	0.555	***
AG_Aaf_422	4	0.588	0.465	ns	5	0.895	0.598	**	5	0.933	0.660	ns
AG_Aaf_434	2	0.923	0.497	**	2	0.100	0.095	ns	2	0.353	0.291	ns

Note

*A* = number of alleles per locus; *H*_e_ = expected heterozygosity; *H*_o_ = observed heterozygosity; HWE = test for deviation from Hardy–Weinberg equilibrium (ns = not significant, *P < 0.05, **P < 0.01, ***P < 0.001); *n* = number of sampled individuals per population.

a Locality and voucher information are provided in Appendix 1.

**Table 3:** Results from cross‐amplification of 15 newly developed microsatellite loci for *Afzelia africana* in two closely related species.^a^

Locus	*Afzelia bipindensis* (*n* = 10)				*Afzelia quanzensis* (*n* = 8)			
	*A*	Allele size range (bp)	*H*_o_	*H*_e_	*A*	Allele size range (bp)	*H*_o_	*H*_e_
AG_Aaf_12	—	—	—	—	4	139–166	0.667	0.583
AG_Aaf_14	—	—	—	—	5	85–99	0.400	0.760
AG_Aaf_15	4	127–142	0.750	0.656	4	127–142	0.167	0.625
AG_Aaf_131	—	—	—	—	2	278–280	0.000	0.278
AG_Aaf_212	3	161–169	0.400	0.560	3	161–175	1.000	0.611
AG_Aaf_214	—	—	—	—	2	189–201	0.833	0.486
AG_Aaf_276	6	85–123	0.600	0.800	3	91–103	0.200	0.340
AG_Aaf_305	2	83–89	1.000	0.500	2	75–89	0.000	0.444
AG_Aaf_350	3	107–113	0.750	0.594	2	113–117	1.000	0.500
AG_Aaf_358	3	91–94	0.250	0.531	1	91	0.000	0.000
AG_Aaf_386	4	228–243	0.000	0.720	2	228–267	0.000	0.278
AG_Aaf_392	6	71–81	0.400	0.820	1	71	0.000	0.000
AG_Aaf_403	1	122	0.000	0.000	2	122–127	0.667	0.444
AG_Aaf_422	3	83–91	0.200	0.460	2	89–91	0.333	0.444
AG_Aaf_434	1	99	0.000	0.000	1	99	0.000	0.000

Note

— = no amplification; *A* = number of alleles per locus; *H*_e_ = expected heterozygosity; *H*_o_ = observed heterozygosity; *n* = number of individuals.

a Locality and voucher information are provided in Appendix 1.

## CONCLUSIONS

The 15 new polymorphic microsatellite markers for *A. africana* reported here will be used for investigation of genetic diversity and structure of populations as well as gene flow in *A. africana*. They will be useful in supporting the conservation and sustainable management of *A. africana* in West Africa.

## Data Availability

Raw sequencing reads were deposited in the National Center for Biotechnology Information (NCBI) Sequence Read Archive (BioProject PRJNA507894; accession number SRP172013). All sequence information of the 15 microsatellite loci was uploaded to the NCBI's GenBank, and the accession numbers are provided in Table 1.

## APPENDIX 1 Voucher information for *Afzelia* samples used in this study.

**Table:**

Species	Collector/collector no.	Collection location	Geographic coordinates (latitude, longitude)	Voucher accession no. (Herbarium)^a^	*n*
*A. africana* Sm.	Houehanou/041	Zone Batia, Pendjari, Benin	10.96295, 1.55314	YH244 (BENIN)	20
	Houehanou/113	Tchatchou, Tchaourou, Benin	9.046452, 2.568065	YH245 (BENIN)	20
	Houehanou/079	Azalimè, Lama, Benin	6.964555, 2.160209	YH246 (BENIN)	20
*A. bipindensis* Harms	Donkpegan A./429	Makalaya‐Pallisco, Cameroon	3.339138, 4.390717	AD364 (BRLU)	1
	Donkpegan A./594	Mayumba, Gabon	−3.268648, 10.830927	AD576 (BRLU)	1
	Donkpegan A./604	Mayumba, Gabon	−3.279478, 10.824923	AD586 (BRLU)	1
	Donkpegan A./553	Bambidie CEB, Gabon	−0.74137, 12.92705	AD636 (BRLU)	1
	Donkpegan A./559	Bambidie CEB, Gabon	−0.694649, 12.910154	AD642 (BRLU)	1
	Donkpegan A./576	Makokou, Gabon	0.912698, 13.672766	AD658 (BRLU)	1
	Franck MONTHE/1640	Cameroon	4.766194, 11.746684	FM1640 (BRLU)	1
	V. BOTIKPO/3507	DRC	3.25413, 20.51784	OH3507 (BRLU)	1
	Donkpegan A./dou‐min1	Mindourou, Cameroon	3.582222, 13.402777	AD590 (BRLU)	1
	Donkpegan A./Dou636	Mindourou, Cameroon	3.58518, 13.35395	AD602 (BRLU)	1
*A. quanzensis* Welw.	VTI‐ITTO/Aqua30	Kwalé, Kenya	−4.178, 39.45451	AD542 (BRLU)	1
	VTI‐ITTO/Aqua50	Kwalé, Kenya	−4.17705, 39.44933	AD562 (BRLU)	1
	VTI‐ITTO/Aqua21	Gede, Kenya	−3.30714, 39.97699	AD533 (BRLU)	1
	VTI‐ITTO/Aqua25	Gede, Kenya	−3.30698, 39.98126	AD537 (BRLU)	1
	VTI‐ITTO /Aqua26	Gede, Kenya	−3.31126, 39.97069	AD538 (BRLU)	1
	Proces P./A10	Lubembe, DRC	−10.92012, 22.534652	AD506 (BRLU)	1
	Proces P./A12	Lubumbe, DRC	−10.92042, 22.532668	AD508 (BRLU)	1
	VTI‐ITTO/Aqua1	Gede, Kenya	−3.27285, 39.98435	AD513 (BRLU)	1

Note

DRC = Democratic Republic of Congo; *n* = number of samples.

a Herbarium abbreviations follow Index Herbariorum (http://sweet​gum.nybg.org/scien​ce/ih).
